# Evaluation of the Timeliness of Psychiatric Consultations

**DOI:** 10.14740/jocmr1809w

**Published:** 2014-05-22

**Authors:** Frederick JP Langheim, Eric Heiligenstein

**Affiliations:** a Dean Health Systems, Madison, WI, USA; b Department of Psychiatry, University of Wisconsin School of Medicine and Public Health, Madison, WI, USA; c Mendota Mental Health Institute, Madison, WI, USA

**Keywords:** Consultation liaison psychiatry, Emergency medicine, Quality improvement

## Abstract

**Background:**

Emergency department (ED) delays have multiple causes and create frustration for patients and staff alike.

**Methods:**

New adult psychiatric ED consultations were studied. Elapsed time between workflow stages was tested as a predictor of total time from triage to disposition. To expedite interviews a one-page form was provided for interested patients to complete before psychiatric evaluation.

**Results:**

Total ED time best correlated with time from rooming to consultation request. Total time was not predicted by time to rooming, or from consultation request to arrival of the psychiatric team.

**Conclusions:**

The intervention appeared to significantly reduce interview times. Variation among physicians regarding protocol for psychiatric consultation requests underscored the importance of standardization in quality improvement efforts.

## Introduction

Emergency department (ED) work-flow depends on countless variables that may include, acuity of preceding and incoming cases, staffing, transfer and transport of admitted patients, consultation delays and custodial room turnover. ED visit lengths steadily increased from 2001 to 2005 correlating with increased diagnostic testing [1]. Patients presenting to EDs for psychiatric reasons may be more sensitive to delays and voice their tolerance uniquely. Simultaneously, psychiatric evaluations may require extensive rapport building and considerable collateral information. On average, mental health related ED visits take 42% longer than other ED visits irrespective of acuity, and can average 8 h [2-4]. Furthermore, psychiatric ED visits are a growing component of total ED workload with 21 visits per 1 000 adults in 2000, a 15% increase since 1992 [5].

To better understand psychiatric ED visit work-flow at the University of Wisconsin Hospitals and Clinics (UWHC), a quality improvement initiative was designed to evaluate the patient experience and test an intervention intended to expedite the visit and improve patient satisfaction. We hypothesized that psychiatric ED visit times would correlate with time spent waiting for the emergency medicine physician, for the psychiatrist, or both. Secondarily, we suspected that patient frustration with waiting is exacerbated by seemingly redundant questions from triage, nursing, ED physicians and consultants. Therefore, a patient form intervention was designed allowing patients to use their wait to provide the psychiatrist with rapidly accessible information upon consultation. Due to lack of completion of pre-intervention satisfaction forms by any patient, the evaluation of patient satisfaction was abandoned as a measure, instead, focusing on total ED time and work-flow.

## Methods

A prospective study of psychiatric ED consultations was conducted from April 16, 2010 to June 28, 2010 at UWHC. Psychiatric residents recorded ER consult medical record numbers and dates and asked the ED physicians to place electronic consult orders. Audit trails were requested of all psychiatric consultation orders placed from the ED for the pre- and post-intervention periods. In this way, the date of consultation, room time, consultation order entry (if available), note stating “psychiatry at bedside” (if available), disposition order entry time, psychiatric note initiation and completion times were collated in a spreadsheet and analyzed using SPSS (SPSS for Windows, version 10.1.0; SPSS, Chicago, IL, USA). To avoid the confound of patients already known to ED and psychiatric providers, only new psychiatric patients were included.

Beginning from June 6, 2010, ED physicians requesting psychiatric consultation were also asked to give patients a double-sided form to voluntarily complete. This form was located and clearly labeled among other forms at all ED physician workstations. The form and plan were reviewed with ED representatives at an ED Clinical Operations meeting. The form provided space for patient initials, age, reason for visit, and current psychiatrist and therapist. Patients could circle individual DSMIV diagnosis, and past and present medications listed by trade and generic name according to class. Space was designated for a brief substance use screening, social history and visit goals including hospitalization. At the Clinical Operations meeting, it was agreed the form would be destroyed by the consultant after use. Upon decision to submit the results for publication, this QI project was reviewed and granted exemption by the UW Health Sciences Minimal Risk Institutional Review Board.

## Results

The pre-intervention population consisted of 65 psychiatric ED consultations: 31 identified through the electronic medical record system (EMR), 40 logged by psychiatric providers (only 6 appeared on both lists). Two individuals were seen 3 times over the study period, and 6 individuals were seen twice. Thirty-eight unique consultations of patients who had had no prior psychiatric evaluations were available for analysis. It was believed that those who had had prior psychiatric consultations would present a significant confound in the form of reduced evaluation time due to the existence of considerable data from prior evaluation in the medical record. Of those 38 new evaluations, one was admitted to family medicine and therefore excluded from further analysis based on confound of medical complexity. Only 2 were identified both by resident logs and by the EMR. Of the remaining 37 encounters, average age was 35.5 ± 16.6 (mean ± SD) range 9 to 81; 24 (65%) were female.

Pearson’s correlation analysis of elapsed times for those 37 pre-intervention encounters found statistically significant correlation between the time from ED rooming to psychiatry consult order entry, and the time from ED rooming to disposition order (n = 15): r = 0.720, P = 0.002. Therefore, the time between ED rooming and the decision to consult psychiatry (using order entry time as a proxy for confirmation of this decision) significantly predicted a large component of the total visit time. There was no significant correlation between ED arrival to rooming and ED Rooming to disposition order (n = 37): r = -0.178, P = 0.291. Delay in being roomed did not predict total visit time. Time of evaluation performed by the ED attending was not an available data point. There was no correlation between time from ED rooming to psychiatric consultation order, and time from order to the “psychiatry at bedside” note (n = 12): r = -0.416, P = 0.178. With fewer data points (12), a longer time to consultation order showed a non-significant trend to more rapid arrival of the consultation service. There was no correlation between the timeliness of arrival of the consult provider and the time to disposition order entry (n = 12): r = -0.137, P = 0.671. A delay in arrival of the psychiatric consultant was not correlated with longer visit times. According to these data, the average pre-intervention time from ED rooming to disposition was 4 h and 54 min (see the table for various times).

According to the post-intervention log, 22 consultations were performed, of which 11 were new to psychiatry. Of those, 5 had EMR consultation orders. One was excluded for requiring a translator. One was excluded due to admission to the family practice service. Of the remaining 9, average age was 26.8 ± 10.6 (mean ± SD) range 12 to 47; 6 (about 67%) were female. These population differences precluded valid statistical comparison between pre- and post-intervention data. When comparing elapsed times in the post-intervention, however, a marked difference was noted in time from consultation order entry to disposition order entry following institution of the patient form: pre-intervention 3:36, post-intervention 2:17 (Table 1). This apparent difference is tempered by essentially equivalent post-intervention time from ED rooming to disposition of 4 h and 50 min. Nevertheless, post study survey of residents found that 100% of those who had used the form had found it helpful. Utilization of the form was not universal as a result of distribution problems secondary to ED providers being unfamiliar with the location of forms and/or time constraints precluding the form being provided to the patient. It is also likely that some patients did not complete the form.

**Table 1:** Elapsed Time Between Stages of Emergency Department Evaluation (hours:minutes)

Average time from	Pre-intervention	Post-intervention
ED room to disposition order	4:54	4:50
ED arrival to ED room	0:24	0:11
ED room to Ψ Consult order	1:25^a^	1:36^b^
Ψ Consult order to Ψ at bedside note	0:56^a^	0:46^b^
Ψ Consult order to disposition	3:36^a^	2:17^b^
Ψ at bedside to disposition order	2:16	2:27

^a^n = 15; ^b^n = 3.

## Discussion

We sought to better understand the factors influencing elapsed time in psychiatric consultations in the UWHC ED. Our data suggest that the time required for initial ED evaluation and consultation request may be the strongest predictor of total visit time. This may reflect ED workload and complexity of cases among other factors. At the same time, provision of a short self-report form may expedite new psychiatric evaluations in the ED.

It is difficult to interpret these results, given the inconsistent documentation of psychiatric consultations to the ED. This is most apparent in the lack of overlap between EMR and resident generated consultation lists, resulting from inconsistent use of EMR consultation order entry. These data suggest a large number of unreported consultations, while those without EMR consultation orders had no documentation of the time of consult request (presumably made by telephone). Even so, entry of disposition orders and signing of notes may be delayed by many variables. For example, a “psychiatry at bedside” note was logged at the same hour and minute the psychiatry resident signed their completed consultation note (these data were excluded from analysis as the patient was not new to the service).

Despite these limitations, it appeared that the time to disposition for psychiatric patients was more closely related to how long it took the ED to consult psychiatry, rather than the time taken for the arrival, assessment and recommendations provided by the psychiatric consultation service. Standardizing electronic consultation orders will allow for more accurate measures. In the future, consideration may be given to comparing these times across other consulting services. Nevertheless, our limited data indicated that a one-page paper form may significantly reduce ED visit times for psychiatric patients.
